# Respiratory infections in the post-COVID-19 era: impact, prevalence, and clinical characteristics of bacterial and viral co-infections

**DOI:** 10.3389/fmed.2025.1597782

**Published:** 2025-10-23

**Authors:** I. Trifonova, N. Korsun, V. Levterova, D. Pavlova, I. Simeonovski, M. Ivanova, P. Velikov, S. Voleva, I. Ivanov, D. Ivanov, T. Dakov, T. Tcherveniakova, S. Angelova, I. Christova

**Affiliations:** ^1^National Laboratory “Influenza and ARD”, Department of Virology, National Centre of Infectious and Parasitic Diseases, Sofia, Bulgaria; ^2^Infectious Disease Hospital “Prof. Ivan Kirov”, Department for Infectious Diseases, Parasitology and Tropical Medicine, Medical University of Sofia, Sofia, Bulgaria; ^3^Clinical Virology Laboratory, University Hospital “Prof. Dr. Stoyan Kirkovich,” Trakia University, Stara Zagora, Bulgaria

**Keywords:** respiratory infections, co-infection, *Streptococcus pneumoniae*, SARS-CoV-2, respiratory virus, clinical characteristics, COVID-19

## Abstract

**Introduction:**

Humans are affected by respiratory infections globally, originating from both bacterial and viral agents. However, the pathogens responsible for respiratory tract infections and the specific effects of viral-viral, viral-bacterial, and bacterial-bacterial co-infections on disease progression and clinical outcomes remain unclear. Тhis study aimed to determine the prevalence, estimate the age burden, and provide clinical characteristics of mono- and co-infections involving various bacterial and viral co-pathogens.

**Methods:**

A total of 609 nasopharyngeal specimens were collected from outpatients and hospitalized patients with respiratory symptoms between April and December 2024. The specimens were analyzed using an in-house multiplex real-time polymerase chain reaction method. Six separate primer and probe mixtures were prepared to detect 15 respiratory viruses and 5 common bacterial respiratory pathogens.

**Results:**

This study, conducted over an 8-month period, found that 65.7% of the patients (400) had at least one respiratory pathogen, with viral infections (49.2%) being more common than bacterial infections (16.5%). Infections were categorized as follows: Viral mono-infections: 217 cases (35.6%); Bacterial-bacterial co-infections: 6 cases (1%); Viral-viral co-infections: 39 cases (6.4%); and Bacterial-viral co-infections: 87 cases (34.3%). Common pathogens causing mono- and co-infections included SARS-CoV-2, rhinovirus, influenza A/B, bocavirus, adenovirus, *Streptococcus pneumoniae*, and *Streptococcus pyogenes*. Mixed infections were more common in children aged <5 years (*p* < 0.05). The disease resulted in a fatal outcome in four patients (1.3%). Notably, one patient with confirmed *Streptococcus pneumoniae* serotype 11A/D had a fatal outcome. Analysis of age as a factor in infection severity revealed that infants aged 7–11 months and patients aged >65 years with bacterial-viral co-infections had mean saturations of 89.6 ± 10.4% and 90 ± 5%, respectively. Moreover, hospitalized patients aged >65 with viral-viral co-infections exhibited significantly higher C-reactive protein levels (150.8 ± 14.3 mg/L) compared to children aged < 15 (*p* < 0.05).

**Conclusion:**

This study identified SARS-CoV-2, rhinoviruses, adenoviruses, and *Streptococcus pneumoniae* as key respiratory pathogens associated with mono- and co-infections. Mixed infections with SARS-CoV-2 were associated with progressively worsening symptoms, particularly in patients aged >65 years. This study highlights the need for preventive measures, including vaccination and revaccination of older adults with vaccines covering a broader range of *S. pneumoniae* serotypes.

## 1 Introduction

Acute respiratory infections are the leading cause of acute illness worldwide, resulting in nearly 4 million deaths annually (1). Millions of people worldwide are affected by respiratory infections yearly, originating from both bacterial and viral agents (2). Bacterial infections often occur secondary to viral infections (3). Viruses are common causes of upper respiratory tract infections (RTIs), while both bacterial and viral agents can lead to lower RTIs (LRTIs) (4). The major viral agents causing RTIs include influenza viruses A and B, respiratory syncytial virus (RSV), human metapneumovirus (HMPV), parainfluenza virus (PIV), adenovirus (AdV), rhinoviruses (RVs), enteroviruses (EVs), and human coronaviruses (HCoV). These viruses can infect airway epithelial cells and utilize host proteins to enhance their infection capability. They can also alter immune responses and trigger inflammation, contributing to the development of diseases (5). The most considerable etiological agents of severe LRTIs include bacteria such as *Streptococcus pneumoniae* (StPn) and *Haemophilus influenza* (HI), as well as viruses like SARS-CoV-2, influenza virus, and respiratory syncytial virus (RSV) (6). A study conducted in Bulgaria during the COVID-19 pandemic found that *Haemophilus influenzae* and RSV were the most common co-pathogens in patients positive for SARS-CoV-2 (7). Our previous studies have also found that the presence of an additional viral or bacterial co-pathogen, especially in patients over 65 years of age, is a risk factor for severe COVID-19 (8). Furthermore, the presence of more than two infectious agents can increase the risk of death by 26%. Viruses are more prevalent in mild upper and middle RTIs and bronchiolitis in children, while bacteria are the primary cause of pneumonia, especially in adults (9). In cases of community-acquired pneumonia, *Streptococcus pneumoniae* is the most commonly identified bacterial agent. Atypical pneumonia may be caused by agents such as *Mycoplasma pneumonia* (MyPn), *Chlamydia pneumonia* (ChPn)*, Legionella, Coxiella burnetii*, and various viruses (10). Nosocomial pneumonia and pneumonia in immunocompromised patients are typically associated with proteinaceous etiologies, predominantly involving gram-negative organisms and staphylococci (11).

Pneumococcal infections are the primary cause of milder RTIs, such as otitis and sinusitis, along with more severe illnesses, including acute bronchitis, COPD, pneumonia (with or without septicemia) and meningitis (12). The introduction of pneumococcal conjugate vaccines in childhood vaccination programs in many countries has considerably reduced the incidence of severe invasive pneumococcal disease in vaccinated children. However, there has been a rise in infections caused by non-vaccine serotypes, which has led to invasive pneumococcal disease in unvaccinated populations, such as older adults. This trend has abated the overall effectiveness of these vaccines (12, 13). A recent national survey in Bulgaria found that the prevalence of *Streptococcus pneumoniae* in children under 6 years of age was 40.8%. This finding may be due to the recent implementation of the PCV10 vaccination strategy in Bulgaria, in which some of the widespread and invasive serotypes were not included in the vaccine (14). S*treptococcus pyogenes* (StPy) is a major bacterium that affects humans, causing a wide range of clinical conditions from mild localized infections to severe life-threatening diseases. It is commonly associated with throat infections, causing 5–15% of cases of pharyngitis in adults and 20–30% of cases in children (15). While some species of the genus Streptococcus are significant human pathogens, most are commensal and part of the normal microbiota found on human skin and mucous membranes. The most notable pathogenic species in this genus are *S. pyogenes*, *S. agalactiae*, and *S. pneumoniae* (16).

Diagnosing the specific cause of RTIs can be challenging, often leading to antibiotic treatment. This approach can result in both inappropriate and appropriate antibiotic use among patients. The clinical syndromes associated with respiratory infections often overlap, with increasing evidence of bacterial-viral co-infections, as well as bacterial pneumonia developing secondary to viral infections (6, 17). Pathogens that co-infect a host can influence one another in various ways. They may compete for essential resources, interfere with each other’s replication, or interact indirectly with the immune system of the host (18). These interactions can alter pathogen transmission, their pathogenicity, and the clinical symptoms and outcomes associated with infections. Furthermore, co-infection can influence epidemic dynamics and affect disease severity and vaccine effectiveness (19). Several factors, including the order in which pathogens appear (20), the timing of infections, and specific pathogen combinations, can synergistically impact disease severity (21).

Monitoring bacterial co-infection dynamics is crucial for ensuring that hospitalized patients receive the most appropriate empirical antibiotic treatment. Implementing multiplex testing for patients with respiratory symptoms enables rapid differentiation between bacterial and viral infections. This approach not only minimizes unnecessary antibiotic use but also allows for more strategic prioritization of treatment options.

Although several studies have investigated the pathogens responsible for RTIs, the specific effects of viral-viral, viral-bacterial, and bacterial-bacterial co-infections on disease progression and clinical outcomes remain unclear. This gap in current research motivated us to further investigate the clinical manifestations of both viral and bacterial infections. This study aimed to determine the causative agents of respiratory infections and assess how the host influences the spread and clinical progression of co-infections involving viral and bacterial respiratory pathogens. This study will contribute to clarifying the clinical outcomes of multiple respiratory infections and ultimately facilitate effective prevention and treatment strategies to improve patient care.

## 2 Materials and methods

### 2.1 Patients and sampling

Between April and December 2024, nasopharyngeal samples were collected from 609 patients presenting with symptoms and signs of bacterial or viral respiratory infections. Samples were obtained from both outpatient and hospitalized patients. This clinical study was conducted in two hospitals in the country: SBALPB “Prof. Ivan Kirov” in Sofia and UMBAL “Prof. Dr. Stoyan Kirkovich” in Stara Zagora. A team of infectious disease specialists and pediatricians analyzed clinical indicators and symptoms based on the patient’s medical history including: blood oxygen saturation (SpO2%), lymphocytes (Lym %), monocytes (Mo %), granulocytes (Gr %), hemoglobin (Hem g/L), leukocytes (WBC 10^9^/L), C-reactive protein (CRP mg/L) in Supplementary Table 1. Blood samples for blood tests were collected only from hospitalized patients at least twice: once on admission and once on discharge. In addition, interim blood tests were performed during longer hospital stays for monitoring purposes. Our study included blood test results only from blood taken on admission from patients with respiratory symptoms. Personal and clinical data were collected from both outpatients and hospitalized patients. These data were part of the information included in a nasopharyngeal swab sample letter sent by general practitioners, medical centers, emergency departments, and hospital units nationwide to the NRL “Influenza and ARD.” These letters typically included: age, gender, and location, and other symptoms of respiratory infections; presence of: fever, headache, diarrhea, runny nose, cough, shortness of breath, etc.; diagnosis: ARI, ILI, and other; and complications, such as bronchiolitis, bronchitis, pneumonia, and other.

The main inclusion criteria for this study were all patients who showed respiratory symptoms, whose samples for respiratory pathogen testing were sent to the NRL “Influenza and ARD.” The inclusion period for individual participants was defined as those who exhibited respiratory symptoms within 10 days of sample collection. All patients studied received the pneumococcal vaccine PCV-10 only during early childhood, after the 6th week of birth.

The study protocol was approved by the ethics committee (IRB 00006384, protocol 5/2022), and informed consent was obtained from all participants or their guardians.

Samples were collected in commercial containers containing viral transport media. These samples were collected during the doctor’s visit or within the first 24 h of admission. After collection, the samples were transported to the NRL “Influenza and ARD” in a refrigerator at 4 °C, within a maximum of 72 h.

### 2.2 Exclusion criteria

Patients exhibiting symptoms that were not typical of respiratory infections were excluded from the study. Additionally, individuals who were unwilling or unable to provide consent were also excluded. Furthermore, any laboratory samples that did not meet the specified criteria for the transportation and storage of nasopharyngeal samples were excluded from the study.

### 2.3 Sample testing

#### 2.3.1 Extraction

Upon arrival at the laboratory, specimens were extracted using an automated extraction system equipped with the Exi-Prep Dx Viral DNA/RNA kit from Bioneer, Daejeon, Republic of Korea. This system isolates viral and bacterial DNA/RNA, producing an eluate of up to 100 μL. Further analysis was performed on the same day, or the eluate was frozen at −20 °C until analysis could be performed.

#### 2.3.2 Multiplex real-time PCR

To simultaneously detect 5 bacterial and 15 viral pathogens, we prepared seven multiplex PCR mixes in the following combinations: MyPn + StPy; ChPn + StPn + HI; AdV + RSV + PIV1; BoV + RV + PIV2; HMPV + PIV3; HCoV-229E + HCoV-HKU-1 + HCoV-NL63 + HCoV-OC43; SARS-CoV-2 + influenza A + influenza B.

The multiplex PCR mix was prepared using the Applied Biosystems™ TaqMan™ Multiplex Master Mix (Thermo Fisher Scientific, Waltham, MA, USA). The primers and probes used for detecting bacterial pathogens are listed in Supplementary Table 2, while those for detecting viral pathogens are reported in another publication, along with the reaction temperature conditions (7). Positive and negative controls were included in each run. For influenza type A and type B viruses, positive controls were provided by IRR, USA; for other viruses, AmpliRun DNA/RNA Amplification Controls (Vircell, Spain) were used. Respiratory pathogens were tested using the QuantStudio™ 5 96-well real-time PCR system (Thermo Fisher Scientific, Waltham, MA, USA). Samples with a cycle threshold (Ct) value of < 38 were considered positive.

#### 2.3.3 *Streptococcus pneumoniae* typing

The samples were screened with the lytA and cpsA genes by real-time PCR. The lytA gene is responsible for producing the protein autolysin, which determines the pathogenesis of *S. pneumoniae* and is a virulence marker. It distinguishes the bacteria from other species such as *S. mitis* and *S. oralis*. The cpsA gene is present in all capsular pneumococci. The presence of a capsule is a criterion for bacteria typing.

The positive samples for both genes underwent PCR and subsequent allelic hybridization using the commercial kit “S. PneumoStrip” (Operon S. A., Zaragoza, Spain), which allows easy and fast identification of 76 different serotypes/serogroups of *S. pneumoniae* in DNA samples with a single amplification. Among these serogroups are those included in the vaccines available to date.

All serotypes/serogroups included were:

Band A: 1, 3, 4, 5, 6A, 6B, 6C, 6D, 7F/7A, 9A/9 V, 14, 18A, 18B/18C, 18F, 19A, 19F, 23A, 23B, and 23\u00B0F.

Band B: 2, 8, 9 N/9 L, 10A, 10B, 10F/C, 11A/D, 11B, 11C, 11F, 12A/46, 12B/44, 12F, 15B/15C, 17F, 20, 22F/22A, 33F/33A, and 37.

Band C: 7B, 7C/40, 15A, 15F, 16F, 19B/19C, 21, 25A/25F, 38, 24A, 24B/24F, 31, 32A/32F, 33B/33D, 33C, 35A, 35C, 35F, 47F, 41A, and 41F.

Only lytA-positive samples are considered non-capsular, and pneumococcus typing was not performed. During hybridization analysis, lytA and cpsA were also determined as control genes.

### 2.4 Definitions

Bronchitis, bronchiolitis, and pneumonia were diagnosed based on chest X-ray results and pulmonary infiltrate presence.

Patient assessment utilized a categorical scale ranging from 1 to 7 to evaluate their clinical status:

1. Not hospitalized, with resumption of normal activities.
2. Not hospitalized, but unable to resume normal activities.
3. Hospitalized, no need for supplemental oxygen.
4. Hospitalized, requiring supplemental oxygen.
5. Hospitalized, requiring high-flow nasal oxygen therapy, noninvasive mechanical ventilation, or both.
6. Hospitalized, requiring extracorporeal membrane oxygenation, invasive mechanical ventilation, or both.
7. Death.

### 2.5 Statistical analysis

The Fisher’s exact tests and chi-square tests were used to analyze categorical variables. For continuous variables, comparisons were made using the Mann–Whitney U test, supported by OriginPro software and GraphPad.^1^ *p*-values < 0.05 were deemed statistically significant.

## 3 Results

### 3.1 Characteristics of the patients studied

This study was included 609 patients (302 hospitalized patients and 307 outpatients; age: 1 month to 97 years [mean: 14.66 ± 22.14 years]; Male: 48.4%, Female 51.5%) who had sought consultation from either their general practitioners or the hospital emergency department because of respiratory infection-related complaints. We further categorized the patients into eight groups based on age: 0–6 months (*n* = 75, 12%), 7–11 months (*n* = 49, 8%), 12–24 months (*n* = 120, 20%), 25–59 months (*n* = 55, 9%), 5–14 years (*n* = 155, 25%), 15–29 years (*n* = 38, 6%), 30–64 years (*n* = 76, 12%), and ≥ 65 years (*n* = 41, 7%).

### 3.2 Etiology of respiratory infections

In this study, conducted over 8 months, respiratory infection with at least one pathogen was observed in 400 (65.7%) patients. The proportion of viral infections (49.2%) was higher than that of bacterial infections (16.5%). Among these infections, 67% (268 cases) were identified as mono-infections, whereas co-infections accounted for 33% (132 cases).

The distribution of confirmed viruses is as follows: SARS-CoV-2 (*n* = 126, 31%), RV (*n* = 97, 24%), BoV (*n* = 49, 12%), influenza A/B (*n* = 49, 12%), AdV (*n* = 33, 8.3%), PIV1 (*n* = 15, 3.8%), PIV3 (*n* = 13, 3.2%), HMPV (*n* = 6, 1.5%), PIV2 (*n* = 5, 1.3%), RSV (*n* = 3, 0.8%), OC43 (*n* = 3, 0.8%), 229E (*n* = 3, 0.8%), and NL63 (*n* = 2, 0.5). No cases of HKU1 infection have been detected. Among the proven bacterial pathogens, streptococci was the most prevalent: StPn (*n* = 84, 21%) and StPy (*n* = 63, 15.8%), while ChPn and MyPn (*n* = 4, 1%; *n* = 1, 0.3%) had significantly lower frequencies among all infected patients.

### 3.3 Prevalence of viral and bacterial mono- and co-infections

The identified infections were categorized by the type of respiratory pathogen—viral or bacterial—and the nature of the co-infections: 51 cases (8.4%); viral mono-infections: 217 cases (35.6%); bacterial-bacterial co-infections: 6 cases (1%); viral-viral co-infections: 39 cases (6.4%); bacterial-viral co-infections: 87 cases (34.3%). Co-detections of two or three co-pathogens were found as bacterial-bacterial, viral-viral, and mixed bacterial-viral infections. In addition, combinations involving four or five co-pathogens were also found among mixed bacterial-viral co-infections. SARS-CoV-2 and RV detection rates were comparable in patients with confirmed mixed viral and bacterial-viral infections. However, influenza A/B, bocavirus, and adenovirus detection rates were significantly higher in patients with mixed viral infections than in those with mixed bacterial-viral infections (*p* = 0.03; *p* = 0.0002; *p* = 0.2209) (Table 1).

**Table 1:** Respiratory viruses, detected in cases of mono and mixed virus-virus and bacterial-virus infections.

	Mono-infections *n* = 217	Viral-viral co-infections *n* = 39	Bacterial-viral co-infections *n* = 87	*p*-value viral-viral vs. bacterial-viral co-infections
Influenza A/B	35 (16)	8 (21)	6 (7)	0.0333
SARS-CoV-2	73 (34)	17 (44)	36 (41)	0.8470
RSV	2 (1)	1 (3)	0 (0)	0.3047
RV	50 (23)	18 (46)	29 (33)	0.2316
BoV	19 (9)	18 (46)	12 (14)	0.0002
PIV1	11 (5)	0 (0)	4(5)	0.3100
PIV2	2 (1)	0 (0)	3(3)	0.5518
PIV3	7 (3)	3 (8)	3(3)	0.3725
HMPV	4 (2)	1 (3)	1 (1)	0.5250
AdV	9 (4)	10 (26)	14(16)	0.2261
OC43	1(0.5)	2 (5)	0 (0)	0.0873
NL63	2 (1)	0 (0)	0 (0)	n.s.*
229E	3(1.4)	0 (0)	0 (0)	n.s.*
HKU-1	0 (0)	0 (0)	0 (0)	n.s.*

n.s.*, non-significant.

*S. pneumoniae* and *S. pyogenes* are common pathogens involved in mixed viral infections. Moreover, *Chlamydia pneumoniae* can be present both in mono- and co-infections involving another respiratory bacterial co-pathogen. The prevalence of each proven bacterial pathogen in various combinations of mixed infections is detailed in Table 2. *Streptococcus pneumoniae* is classified into two: noncapsular and capsular, as detailed in Table 3. A significant majority, 95%, of *S. pneumoniae* were found to be capsular, whereas only 5% were noncapsular. Nineteen different serotypes of capsular *S. pneumoniae* were documented: 3, 6 AC, 6C, 9 N/9 L, 10A, 10F, 12A/46, 11A/D, 15B/15C, 15A/15F, 19A, 19B/19C, 20, 22F/22A, 23A, 23B, 23F, 24A/24B/24F, and 33F/33A. However, we were unable to determine the serotype for four of the identified *S. pneumoniae* cases. Twelve patients infected with capsular *Streptococcus pneumoniae* were co-infected with two different serotypes.

**Table 2:** Distribution of proven respiratory bacterial pathogens based on their presence in mono- and co-infections with a viral or other bacterial co-pathogen.

	StPn, *n* = 84	StPy, *n* = 63	ChPn, *n* = 4	MyPn, *n* = 15	HI, *n* = 1
Mono-infection, *n* (%)	24 (28.6)	21 (33.3)	2 (50)	6 (40)	0 (0)
Virial co-infection, *n* (%)	55 (65.5)	39 (62)	1 (25)	5 (33.3)	1 (100)
Bacterial co-infection, *n* (%)	5 (5.9)	3 (4.7)	1(25)	4 (26.7)	0 (0)

**Table 3:** Distribution of confirmed *S. pneumoniae* based on their classification as non-capsular or capsular, along with the distribution of the corresponding serotypes of capsular *Streptococcus pneumoniae* categorized directly into eight age groups for this study.

	Nonencapsulated	Capsular serotypes of Streptococcus pneumoniae*																										Total
		3	3;15B/15C	6 AC	6C	6C;15A/15F	6C;15B/15C	6C;19A	10A	10A;12A/46	10F/C;15B/15C	10F/C;19B/19C	10F/C;19B/19C	11A/D	15B/15C	15B/15C;19B/19C	19A	19A;6C	19B/19C	20	22F/22A	23A;19B/19C	23B	23F	33F/33A	24A/24B/24F;9N/9 L	Not serotyped (for use kit in this study)	
Age group**																												
0–6 months *n* = 75		1		1	2					1				1	1										1		1	9
7–11 months *n* = 49		1			2				1						1			1			1	1		1			1	10
12–24 months *n* = 120		1			1	1								3	3		7		1				2				1	20
25–59 months *n* = 55	1		1		1							1		2		1	2						1					10
5–14 years *n* = 155	2						1	1	1		1		1				1			1						1	1	11
>65 years *n* = 41														1														1
Total	3	3	1	1	6	1	1	1	2	1	1	1	1	7	5	1	10	1	1	1	1	1	3	1	1	1	4	61
Bacterial co-pathogen																												
MyPn, StPn												1																1
MyPn, StPn, Ch. Pn.	1																											1
St. Pyog, StPn		1									1			1	1		3			1					1			9
StPn, HI				1																								1
StPn, MyPn								1																				1
StPn	2	2	1		6	1	1		2	1			1	6	4	1	7	1	1		1	1	3	1		1	4	48
Total	3	3	1	1	6	1	1	1	2	1	1	1	1	7	5	1	10	1	1	1	1	1	3	1	1	1	4	61
Viral co-pathogen																												
AdV											1			1													1	3
AdV, PIV2, RV			1																									1
AdV, RV,ОС43																	1											1
BoV												1																1
PIV1															1													1
PIV1, BoV														1														1
PIV2					1																							1
PIV3, BoV																									1			1
RV		1		1	1									2		1	1	1		1			2				1	12
RV, BoV					1																							1
RV, AdV															1													1
RV, BoV, AdV															1													1
SARS-CoV-2		1			1				1	1				2	2		2				1			1				12
SARS-CoV-2, AdV																	1											1
*StPn*	2												1	1			2		1							1	2	8
Total	3	3	1	1	6	1	1	1	2	1	1	1	1	7	5	1	10	1	1	1	1	1	3	1	1	1	4	61

0–6 months, 7–11 months, 12–24 months, 25–59 months, 5–14 years, 15–29 years, 30–64 years, and >65 years, presence of additional bacterial respiratory co-pathogen or viral respiratory co-pathogen. Distribution of some *S. pneumoniae* that were not serotyped due to limitations in the commercial kit used (see materials and methods) was also assigned to the categories above (“;” separates one serotype of *S. pneumoniae* from another in the presence of mixed infection with two serotypes). *PCV10 vaccine targets the following ten pneumococcal serotypes: 1, 4, 5, 6B, 7F, 9 V, 14, 18C, 19F, and 23F. **Age groups 15–29 years and 30–64 years are excluded from this table because there were no positive samples for *S. pneumoniae* in these age ranges.

**Figure 1:**
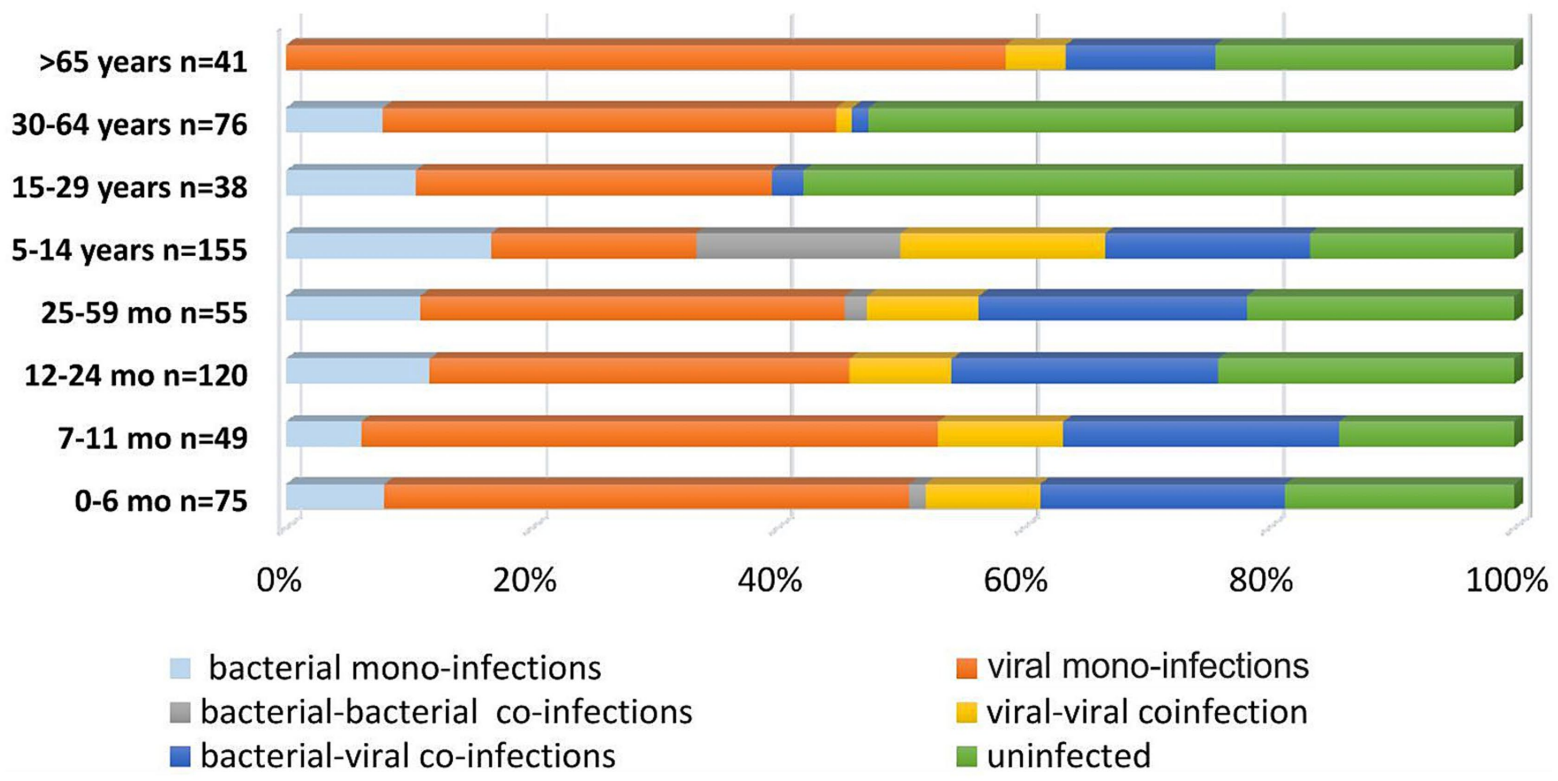
Proportion of respiratory pathogens in mono-bacterial or viral infections, as well as in viral-viral, bacterial-bacterial, and viral-bacterial co-infections.

**Figure 2:**
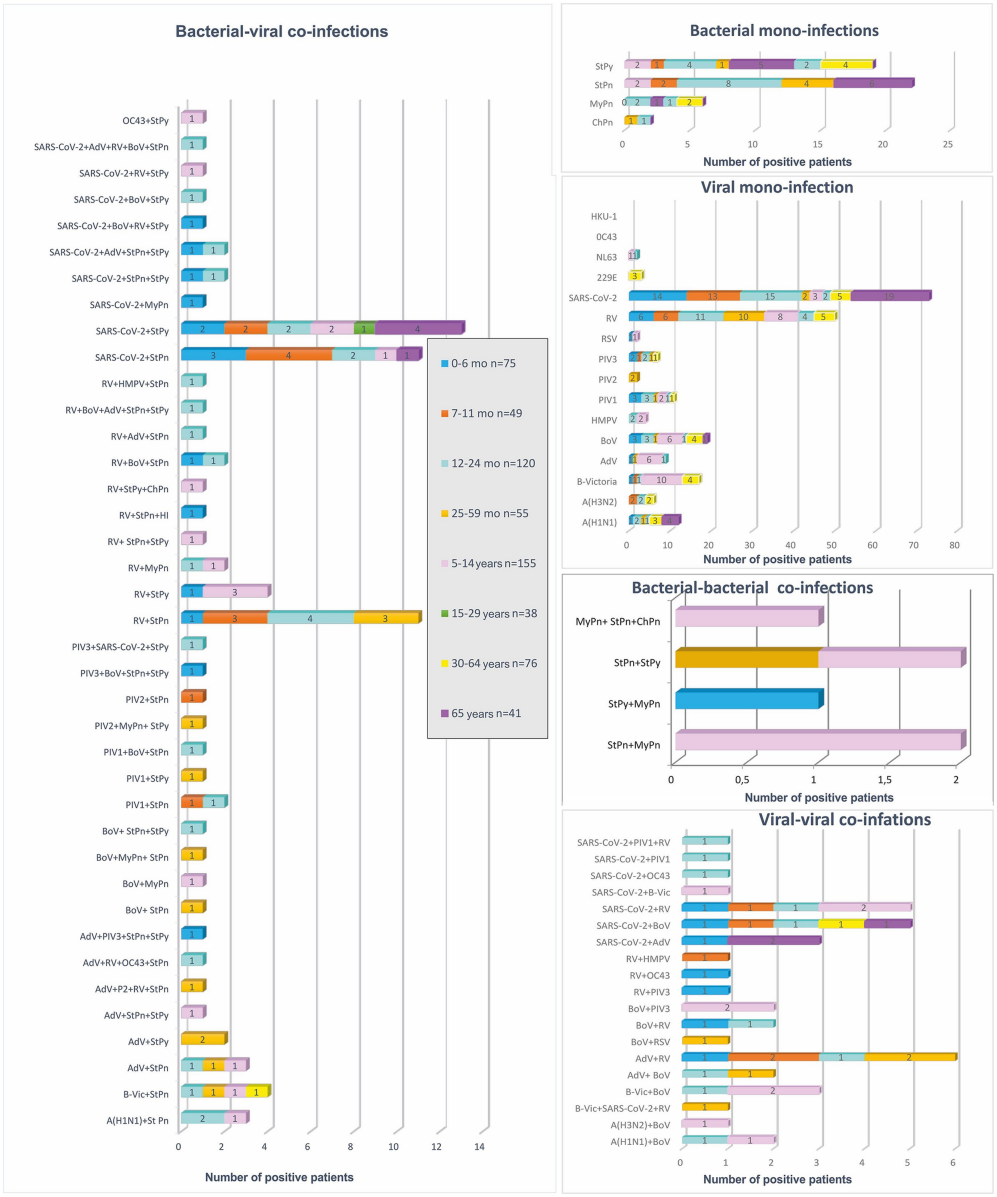
Percentage distribution of confirmed respiratory pathogens categorized by different combinations: bacterial mono-infections, viral mono-infections, bacterial-bacterial co-infections, viral-viral co-infections, and bacterial-viral co-infections. This includes the prevalence of 16 proven respiratory viruses, such as influenza viruses A and B (A/H1N1, A/H3N2, and B/Victoria), respiratory syncytial virus (RSV), rhinovirus (RV), human metapneumovirus (HMPV), parainfluenza virus (PIV) types 1, 2, and 3, adenovirus (AdV), bocavirus (BoV), as well as endemic human coronaviruses. (OC43; 229E; NL63) and 5 bacterial pathogens *Streptococcus pneumoniae* (StPn), *S. pyogenes* (StPy), *Haemophilus influenzae* (HI), *Mycoplasma pneumoniae* (MyPn), and *Chlamydia pneumoniae* (ChPn).

To analyze differences in the distribution of confirmed mono- and co-infections, patients were categorized into eight age groups: 0–6 months (*n* = 75), 7–11 months (*n* = 49), 12–24 months (*n* = 120), 25–59 months (*n* = 55), 5–14 years (*n* = 155), 15–29 years (*n* = 38), 30–64 years (*n* = 76), and ≥65 years (*n* = 41) (Figure 1). Statistical analysis revealed that mixed infections involving both bacterial and viral pathogens were more prevalent in children <5 years than in those aged 5–14 years, with rates of 21.4 and 10.3%, respectively (*p* = 0.0028). In patients > 65 years old, the rate of bacterial-viral co-infections was 12%, comparable to the 10% observed in children and adolescents aged 5–14 years. However, this rate is significantly higher than those found in adolescents and adults aged 15–29 years (2.6%), and in adults aged 30–64 years (1.3%) (*p* = 0.2026; *p* = 0.0339). The age distribution of viral-viral co-infections shows trends similar to those of viral-bacterial co-infections in different age groups. The highest percentage of viral-viral co-infections was in children aged <5 years (9%), followed by persons aged>65 years (6.4%) and children and adolescents aged 5–14 years (5.8%). In contrast, no mixed infections were found in the 15–29 years age group, whereas the 30–60 years age group had mixed infections, although with a significantly lower percentage (1.3%). These data highlight the notable difference compared with the group of children aged <5 years (*p*-values: 0.0559, 0.0248). In this study, no significant differences were observed in the distribution of bacterial and viral co-infections versus mono-infections across the four age groups: 0–6, 7–11, 12–24, and 25–59 months. However, the highest proportion of mixed infections was observed in infants aged 7–11 months (12%) and patients > 65 years (12%), especially those with SARS-CoV-2 combined with another bacterial co-pathogens. Further, infants aged 0–6 months had the highest proportion of mixed infections with SARS-CoV-2 and other viral and bacterial co-pathogens (2.6%) compared with the other age groups. Mixed infections involving AdV and a bacterial co-pathogen, as well as mixed infections with other viral and bacterial co-pathogens, were also observed in children aged <5 years. Children aged 25–59 months had the highest percentage of such mixed infections (2.7%) (Figure 2).

### 3.4 Clinical manifestation of respiratory infection

In this study involving 403 patients infected with at least one respiratory pathogen, 70% reported experiencing symptoms in the letter that accompanied their specimens for testing. The most commonly observed symptoms were fever (50%, 203 cases), cough (48%, 196 cases), runny nose (29.5%, 119 cases), diarrhea (17.6%, 71 cases), vomiting (16.3%, 66 cases), sore throat (7.7%, 31 cases), fatigue (3.7%, 15 cases), headache (1.5%, 6 cases), and shortness of breath (1.2%, 5 cases). Furthermore, among the patients who had a fever, seven experienced febrile seizures.

In comparing viral and bacterial infections, we observed a statistically significant difference between the two symptoms. Diarrhea occurred more frequently in bacterial infections, affecting 30% of patients, compared to 14.8% in viral infections (*p* = 0.0076). Furthermore, sore throat was more prevalent in viral infections, reported by 9.7% of patients, while only 1.7% of those with bacterial infections experienced this symptom (*p* = 0.0376). Among the 285 patients for whom we had information on symptom onset, those who had respiratory symptoms for < 5 days were more likely to have been infected with a viral mono-infection (46.6%) compared with those whose symptoms lasted > 10 days (*p* = 0.05). Notably, the source of infection was more often identified by PCR in patients who had symptoms in the first 5 days (75%), unlike those whose symptoms began after this period (*p* = 0.35; *p* = 0.04). Co-infection with three pathogens was confirmed more frequently in patients who experienced symptoms for > 10 days. In contrast, patients with symptoms lasting up to 10 days were more likely to have co-infection with two pathogens, as shown in Table 4.

**Table 4:** Analysis of proven mono-infections and co-infections, determining the relationship between the number of participants with mixed infections and the duration of respiratory symptoms until the day of the patient’s examination.

	Patients presenting with symptoms of respiratory infection lasting:				
	> 10 days/ *n* = 24	5–10 days/*n* = 103	<5 days/ *n* = 161	*p*-value > 10 days vs. 5–10 days	*p*-value >10 days vs. < 5 days
Bacterial mono-infections, *n* (%)	3 (12.5)	7 (6.8)	11 (6.8)	0.3982	0.3983
Viral mono- infections, *n* (%)	6 (25)	40 (38.8)	75 (46.6)	0.2443	0.0504
Bacterial-bacterial co-infections, *n* (%)	0 (0)	1 (1)	0 (0)	n.s.*	n.s.*
Viral-viral co-infections, *n* (%)	1 (4.2)	7 (6.8)	12 (7.5)	n.s.*	n.s.*
Bacterial-viral co-infections, *n* (%)	3 (12.5)	12 (11.7)	23 (14.3)	n.s.*	n.s.*
Total infections, *n* (%)	13 (54.2)	67 (65)	121 (75)	0.3532	0.0479
Co-infection with two pathogens, *n* (%)	2 (8.3)	16 (15.5)	29 (18)	0.5221	0.3789
Co-infection with three and more pathogens, *n* (%)	2 (8.3)	2 (1.9)	3 (1.9)	0.1615	0.1264
Co-infection with four pathogens, *n* (%)	0 (0)	1 (1)	3 (1.9)	n.s.*	n.s.*
Co-infection with five pathogens, *n* (%)	0 (0)	1 (1)	0 (0)	n.s.*	n.s.*

n.s.*, non-significant.

### 3.5 Clinical characterization of study patients

We conducted a clinical review involving 324 hospitalized patients admitted for respiratory infections, with 321 reporting having at least one symptom typically associated with respiratory disease. The most commonly observed symptoms were as follows: Cough: 180 patients (56%); Fatigue: 174 patients (54%); Decreased appetite: 129 patients (40%); Fever: 116 patients (36%); Runny nose: 109 patients (33.9%); Diarrhea: 102 patients (31.7%); Sore throat: 51 patients (15.8%); Vomiting: 46 patients (14.3%); Headache: 43 patients (13.4%); Shortness of breath: 39 patients (12.1%); and Febrile seizures: 12 patients (3.7%). A higher proportion of patients with mono-infections by bacterial respiratory pathogen had cough and headache than those with bacterial-viral co-infections (Table 5) (*p* = 0.0013 and *p* = 0.0596).

**Table 5:** Clinical manifestations, treatment, and clinical adverse reactions in patients with mono-bacterial infections, mono-viral infections, bacterial-bacterial co-infections, viral-viral co-infections, and bacterial-viral co-infections.

	Bacterial mono-infections	Viral mono-infections	Bacterial-bacterial co-infections	Viral-viral co-infection	Bacterial-viral co-infections	*p*-value bacterial mono-infections vs. viral mono-infections	*p*-value bacterial mono-infections vs. bacterial-viral co-infections	*p*-value viral mono- infections vs. viral-viral co-infection	*p*-value viral mono- infections vs. bacterial-viral co-infections
Distribution (*n*)	39	123	3	20	76	–	–	–	–
With clinical data (*n*)	37	114	3	17	72	–	–	–	–
Symptoms									
Fatigue *n* (%)	18 (48.6)	69 (60.5)	1 (33.3)	8 (47.1)	41 (56.9)	0.2494	0.4252	0.3049	0.6486
Cough *n* (%)	14 (37.8)	65 (57)	2 (66.7)	13 (76.5)	44 (61.1)	0.0577	0.0013	0.1855	0.6475
Diarrhea *n* (%)	15 (40.5)	29 (25.4)	3 (100)	5 (29.4)	23 (31.9)	0.0966	0.4016	0.7692	0.4020
Headache *n* (%)	5 (13.5)	18 (15.8)	0 (0)	2 (11.8)	4 (5.6)	n.s.*	0.0596	n.s.*	0.1139
Rheum *n* (%)	10 (27)	46 (40.4)	0 (0)	8 (47.1)	24 (33.3)	0.1728	0.6628	0.6082	0.3554
Fever *n* (%)	12 (32)	51 (44.7)	0 (0)	6 (35.3)	29 (40.3)	0.2497	0.5319	0.6021	0.6485
Body temperature, mean °C (SD)	38.3 (0.9)	38.1 (0.7)	37.2 (0.6)	37.9 (0.8)	38.1 (0.7)	n.s.*	n.s.*	n.s.*	n.s.*
Complication									
Respiratory tract complication *n* (%)	8 (21.6)	31 (27.2)	2 (66.7)	5 (29.4)	16 (22.2)	0.6659	n.s.*	n.s.*	0.4919
Bronchiolitis *n* (%)	4 (10.8)	16 (14)	1 (33.3)	3 (17.6)	4 (5.6)	0.7830	0.4399	0.7133	0.0893
Pneumonia *n* (%)	5 (13.5)	15 (13.2)	1 (33.3)	2 (11.8)	12 (16.7)	n.s.*	0.7847	n.s.*	0.5274
Nervous system complications *n* (%)	4 (10.8)	6 (5.3)	0 (0)	0 (0)	1 (1.4)	0.2606	0.0444	0.5932	0.2513
Laboratory results									
SpO2, mean % (SD)	93.6 (5.9)	93.2 (5.5)	95.3 (2.2)	94.1 (4.1)	93.6 (5.2)	n.s.*	n.s.*	n.s.*	n.s.*
Hematocrit mean, % (SD)	125.2 (15.6)	120.84 (17.2)	134 (5.1)	115.8 (21.7)	121.4 (12.1)	n.s.*	n.s.*	n.s.*	n.s.*
Lym, mean % (SD)	25.6 (15.9)	32.2 (19.8)	13.9 (7.7)	32.9 (20.7)	30.9 (19.1)	n.s.*	n.s.*	n.s.*	n.s.*
WBC, mean x10^9^/L (SD)	9.6 (4.2)	9 (9)	13.3 (4.3)	10.8 (5.8)	10.2 (5.8)	n.s.*	n.s.*	n.s.*	n.s.*
Mо, mean % (SD)	6.1 (8.1)	6.8 (6.8)	2.9 (1.1)	7.9 (6.3)	6.8 (5)	n.s.*	n.s.*	n.s.*	n.s.*
Gran, mean % (SD)	59.5 (22)	64.9 (22.9)	83.2 (6.7)	59.2 (23.9)	60.8 (22.2)	n.s.*	n.s.*	n.s.*	n.s.*
CRP, mean mg/L (SD)	50.2 (67.8)	40.7 (71)	25.4 (35.4)	53.1 (53.6)	49.6 (87.8)	n.s.*	n.s.*	n.s.*	n.s.*
Treatment									
Antibiotics *n* (%)	15 (40.5)	35 (30.7)	2 (66.7)	4 (23.5)	20 (27.8)	0.3161	0.1984	0.7769	0.7436
Antiviral drugs *n* (%)	0 (0)	2 (1.8)	0 (0)	0 (0)	4 (5.6)	n.s.*	0.2974	n.s.*	n.s.*
Corticosteroids *n* (%)	3 (8.1)	13 (11.4)	0 (0)	2 (11.8)	11 (15.3)	0.7620	0.3742	n.s.*	0.5010
Oxygen therapy *n* (%)	0 (0)	12 (10.5)	0 (0)	1 (10.5)	6 (8.3)	0.0391	0.0938	n.s.*	0.8001
Clinical outcome									
Hospital stay, mean days (SD)	5 (7.1)	3.9 (2)	4.3 (1.15)	3.2 (1.6)	4.1 (1.9)	n.s.*	n.s.*	n.s.*	n.s.*
Duration of illnesses, mean number of days (SD)	6.1 (6.4)	4.8 (7)	1.5 (0.7)	5.1 (7.3)	4.1 (3.6)	n.s.*	n.s.*	n.s.*	n.s.*
Fatal outcome *n* (%)	0 (0)	2 (1.8)	0 (0)	1 (10.5)	1 (1.4)	0.0210	n.s.*	0.3582	n.s.*

n.s.*, non-significant.

Respiratory infection-related complications were also observed, including pneumonia (46 cases), bronchitis (1 case), bronchiolitis (14 cases), and central nervous system complications (21 cases). Some of the patients hospitalized for respiratory infection also had comorbidities such as obesity (*n* = 1/44 years), arterial hypertension (*n* = 23/44–97 years), immunosuppression (*n* = 7/17–45 years), cancer (*n* = 9/26–88 years), diabetes (*n* = 8/44–92 years), chronic obstructive pulmonary disease (COPD). (*n* = 4/64–88 years), neurological (*n* = 8/2–67 years), renal disease (6 months), liver disease (*n* = 2/71–64 years), anemia (*n* = 8/6 months–45 years), and other comorbidities (*n* = 10/7 months–92 years). Nervous system complications (cerebral edema) are more likely to arise from mixed bacterial-viral respiratory infections than from mono-bacterial infections (*p* = 0.0444) (Table 5).

The disease resulted in a fatal outcome in four patients (1.3%). All the deceased patients were aged >65 years and had arterial hypertension. In addition, two of the patients had diabetes, one had chronic lung disease, and one had chronic kidney disease. All patients were infected with SARS-CoV-2. Notably, mono-viral infections were linked to higher mortality rates than mono-bacterial infections, with no deaths reported from the latter (*p* = 0.02). Among them, two individuals had concomitant infections: one was infected with both SARS-CoV-2 and adenovirus, while the other had SARS-CoV-2 in association with *Streptococcus pneumoniae*.

Clinical and laboratory data were available for 44 (69%) patients with confirmed serotypes of capsular *S. pneumoniae* out of 64 patients who tested positive for this pathogen (Table 6). One non-capsular *S. pneumoniae* was identified among the hospitalized patients, although measured CRP and saturation levels were within normal limits (CRP < 10 mg/mL; SpO2 > 94%). Among the confirmed serotypes of capsulated *S. pneumoniae*, two infected patients (5%) had both CRP levels and body temperatures > 100 mg/L and 38 °C, respectively; these serotypes were StPn: 3 (33.3% of 3 cases) and StPn: 11A/D (22% of 5 cases). Further, two of the patients with serotypes StPn: 3 and StPn: 11A/D (40%) had oxygen saturations < 90% and CRP levels > 100 mg/L. One SARS-CoV-2 positive patient, confirmed to have StPn serotype 11A/D, had a fatal outcome, representing 100% of hospitalized StPn-positive patients who experienced a fatal outcome. Assessment of paraclinical data in patients of different age groups.

**Table 6:** Distribution of proven capsular serotypes of *Streptococcus pneumoniae* in patients with the following clinical events and clinical-laboratory data: fatal outcome and CRP > 100 mg/; body temperature >38 °C; SpO2 < 90%.

Capsular serotypes of *Streptococcus pneumoniae*			
Fatal outcome	CRP > 100 mg/L	Body temperature > 38 °C	SpO2 < 90%
11A/D	3	11A/D	10A
	11A/D	23A;19B/19C	3
	3;15B/15C	15B/15C	
	11A/D	10A;12A/46	
		10F/C;15B/15C	
		22F/22A	
		19A	
		6C	

**Table 7:** Distribution of the average measured values of blood parameters in patients with confirmed mixed bacterial-viral infections: saturation (SpO2%), white blood cells (WBC 10^9/L), lymphocytes (Lym%), monocytes (Mo%), hemoglobin g/L and C-reactive protein (CRP mg/L) in the following 8 age groups: 0-6 months, 7-11 months, 12-24 months, 25-59 months, 5-14 years, 15-29 years, 30-64 years, and under 64 years.

Age group	SpO2%, Mean	SpO2, SD	WBC 10^9/L, Mean	WBC 10^9/L, SD	Lym %, Mean	Lym %, SD	Mo %, Mean	Mo %, SD	Hemoglobin g/L, Mean	Hemoglobin g/L, SD	CRP mg/L, Mean	CRP mg/L, SD
0–6 months	93.0	3.7	10.0	6.3	47.2	13.6			112.5	11.6	13.0	15.2
7–11 months	89.6	10.4	12.6	5.6	43.5	24.7	9.7	5.1	115.2	4.4	30.8	63.3
12–24 months	95.1	3.0	9.1	5.8	34.7	16.2	5.8	2.6	120.1	11.6	22.0	24.8
25–59 months	95.5	2.2	12.2	7.2	26.1	20.2			122.9	13.0	58.4	70.2
5–14 years	94.7	3.0	7.6	4.0	17.2	8.4	7.9	7.4	130.4	9.1	32.7	57.8
15–29 years	95.2	–	8.2	–	57.8	–	15.6	–	139.0	–	29.8	–
30–64 years	89.6	–	8.7	–	7.0	–	2.3	–	121.0	–--	171.9	–
<64 years	90.1	5.1	13.6	4.8	10.7	8.0	3.1	2.4	131.0	8.7	206.6	181.9

#### 3.5.1 Age group 0–6 months

**Figure 3:**
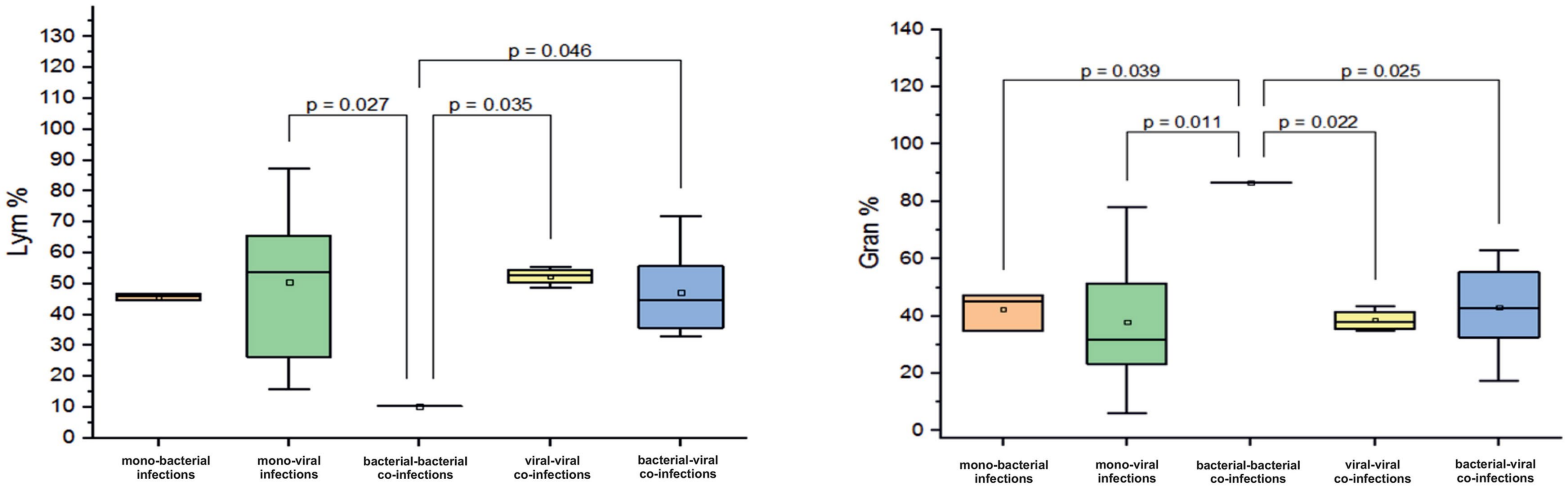
Comparison of the mean values of blood parameters: Lym (Lym%) and the granulocytes (Gran%) levels in infants aged 0–6 months with different categories of infections: mono-bacterial infections, mono-viral infections, bacterial-bacterial co-infections, viral-viral co-infections, and bacterial-viral co-infections. Mean Lym% and Gran% values, median, and range 25–75% are shown (see Lym% and Gran% distribution). Values were calculated using the Mann–Whitney U test (▫ Mean; −Median Line; ┬Range within 1IQR; **∙** Outliers; ┐┌ Fisher LSD).

Co-infection with two bacterial pathogens was detected in one infant aged 0–6 months, resulting in a pronounced lymphopenia of 10.3% and granulocytosis of 86.6%. However, among other infants in this age group solely infected with a bacterial pathogen, the mean Lym (45% ± 0.9) and granulocyte (Gran, 42.4% ± 6.5%) levels were normal. Normal levels of these two blood markers were also found in other infected infants. For instance, those with viral mono-infections had a mean Lym of 50.6% ± 20.4% (*p* = 0.027) and a Gran of 37.9% ± 21%. Infants with viral-viral co-infection had a mean Lym of 52.4% ± 2.8% (*p* = 0.035, Figure 3) and a Gran of 38% ± 3.8%. Those with bacterial-viral co-infection had a mean Lym of 47 ± 13.6% (*p* = 0.046, Figure 3) and a Gran of 43 ± 14.4%.

Further, among infants with a bacterial-bacterial mixed infection, the percentage of monocytes (Mo) was normal at 3% (normal Mo level for infants aged 0–6 months ranges between 1 and 6%). Infants with confirmed mono-bacterial infections had higher mean Mo levels (7.7% ± 1.4%). Similarly, those with monoviral infections, viral-viral co-infections, and bacterial-viral infections had mean elevated Mo levels of 8 ± 4.7%, 8.9 ± 5.2%, and 9.7 ± 5.1%, respectively.

#### 3.5.2 Age group 7–11 months

**Figure 4:**
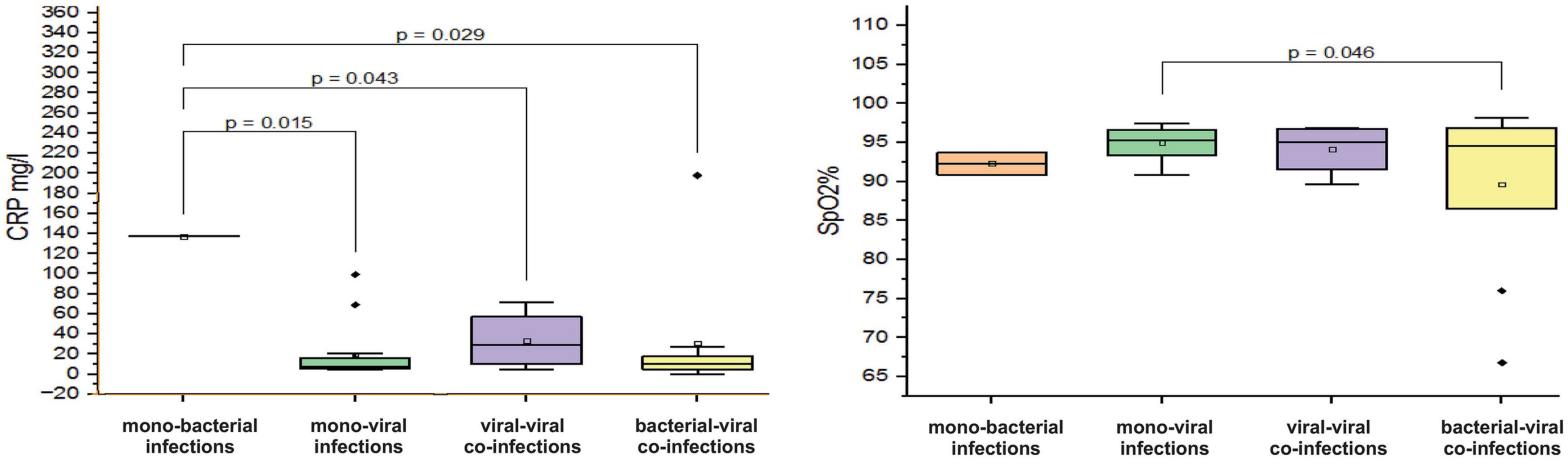
Comparison of the mean values of blood parameters: saturation level (SpO2%) and C-reactive protein (CRP, mg/L) levels in infants aged 7–11 months with different categories of infections: mono-bacterial infections, mono-viral infections, bacterial-bacterial co-infections, viral-viral co-infections, and bacterial-viral co-infections. Mean SpO2% and CRP mg/L values, median, and range 25–75% are shown (see SpO2% and CRP distribution). Values were calculated using the Mann–Whitney U test (▫ Mean; −Median Line; ┬Range within 1IQR; ♦ Outliers; ┐┌ Fisher LSD).

A critically low mean saturation level (SpO2%) of 89.6 ± 10.4% was observed in children aged 7–11 months with confirmed mixed infections involving both bacterial and viral pathogens. In contrast, those who were mono-infected with either a bacterial or viral pathogen had mean SpO2% levels of 92.3 ± 2 % and 94.9 ± 1.9%, respectively (*p* = 0.046, Figure 4). Children with virus-virus co-infections had SpO2% levels closer to normal (94.7± 3.3%). Among patients with viral mono and co-infection, elevated mean Mo levels were observed (mean Mo 9.3 ± 3.3% and 9.7 ± 8.6%). In contrast, patients with mixed bacteria-virus co-infections showed Mo levels within the normal range (5.7 ± 2.5%). In infants aged 7–11 months, a sharp increase in mean CRP levels was observed during bacterial mono-infection, with levels reaching 137.2 mg/L, which is ten times higher than the normal range of 0–5 mg/L. Furthermore, these CRP levels were significantly elevated compared to the mean measured CRP levels in patients with viral mono-infection, virus-viral co-infection, and bacterial-viral co-infection (*p* < 0.05, Figure 4).

#### 3.5.3 Age group 12–24 months

**Figure 5:**
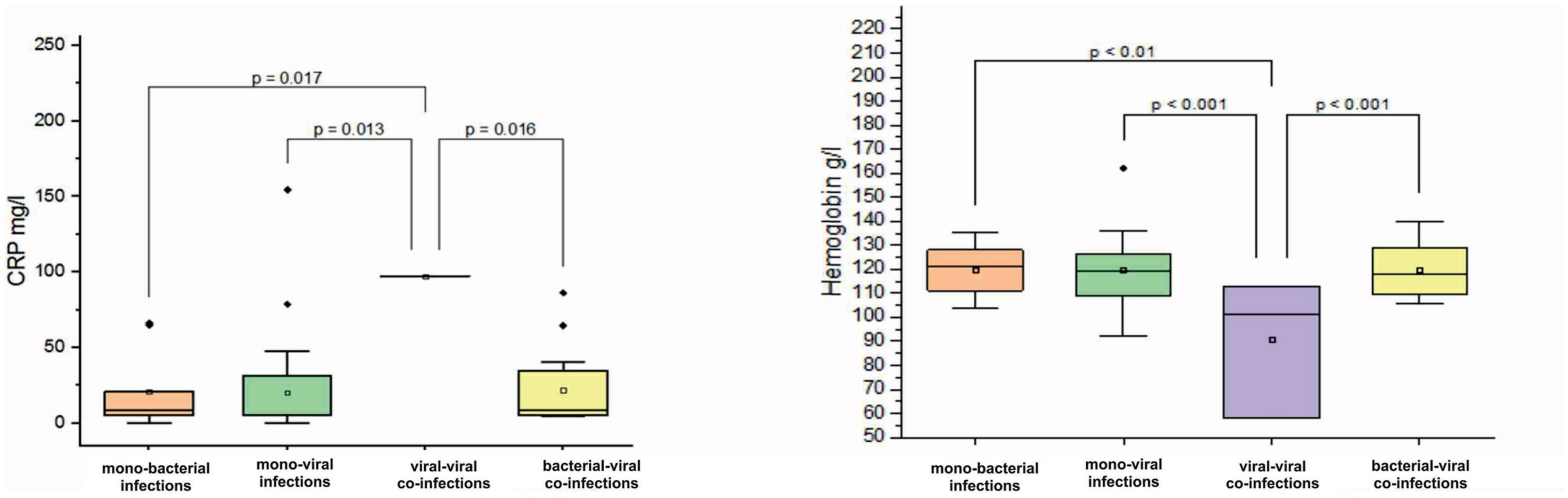
Comparison of mean values of blood parameters: C-reactive protein (CRP, mg/L) and hemoglobin (g/L) levels in children aged 12–24 months with different categories of infections: mono-bacterial infections, mono-viral infections, bacterial-bacterial co-infections, viral-viral co-infections, and bacterial-viral co-infections. Mean CRP mg/L and hemoglobin g/L values, median, and range 25–75% are shown (see CRP mg/L and hemoglobin g/L distribution). Values were calculated using the Mann–Whitney U test (▫ Mean; −Median Line; ┬Range within 1IQR; ♦ Outliers; ┐┌ Fisher LSD).

In children aged 12–24 months, a decrease in hemoglobin was observed in viral–viral co-infections with a mean value of 90.6 ± 28.9 g/L (normal 120–180 g/L) (Figure 5). In contrast, hemoglobin levels in viral mono-infections, bacterial mono-infections, and bacterial–viral mixed infections remained within normal limits with the following mean values: 119.6 ± 13.7 g/L (*p* < 0.001), 119.6 ± 10.3 g/L (*p* < 0.01), and 120.1 ± 11.6 g/L (*p* < 0.001), see Figure 5. In addition, a tenfold increase in CRP levels was observed in viral–viral mixed infections, reaching a mean value of 97.2 mg/L. In comparison, the mean increase in CRP levels for viral mono-infections, bacterial mono-infections, and bacterial-viral co-infections was only twofold: 20.4 ± 32.5 mg/L (*p* = 0.013), 20.8 ± 26 mg/L (*p* = 0.017), and 21.9 ± 24.8 mg/L (*p* = 0.016), see Figure 5.

#### 3.5.4 Age group 25–59 months

In children aged 25–59 months, no significant differences in laboratory and clinical parameters were observed between infection types. However, in this age group, bacterial mono-infections (mean CRP 66.6 ± 80.6 mg/L) and one reported viral co-infection (CRP 52.3 mg/L), as well as mixed bacterial-viral co-infections (mean CRP 58.4 ± 70.2 mg/L), demonstrated approximately five- to six-fold CRP increase. In contrast, a twofold increase in this parameter was observed in proven viral mono-infections (mean CRP 22.8 ± 35.8 mg/L). Critical mean blood oxygen saturation levels of 87.6 ± 15.5% have been reported for bacterial mono-infections. In viral-viral co-infections, a decrease in hemoglobin and Lym by 117g/L and 10.2%, respectively, was observed, while an increase in leukocytes by 11.5 10^9/L was reported. In the remaining combinations of proven mono- and co-infections, the values of hemoglobin and Lym were reported within the normal range (hemoglobin: 120–180 g/L; Lym: 20*–*40%). In mono-bacterial and bacterial-viral mixed infections, a slight increase in leukocytes was reported (mean white blood cell (WBC): 11.9 ± 7.4 10^9/L; 12.2 ± 7.2 10^9/L).

#### 3.5.5 Age group 5–14 years

**Figure 6:**
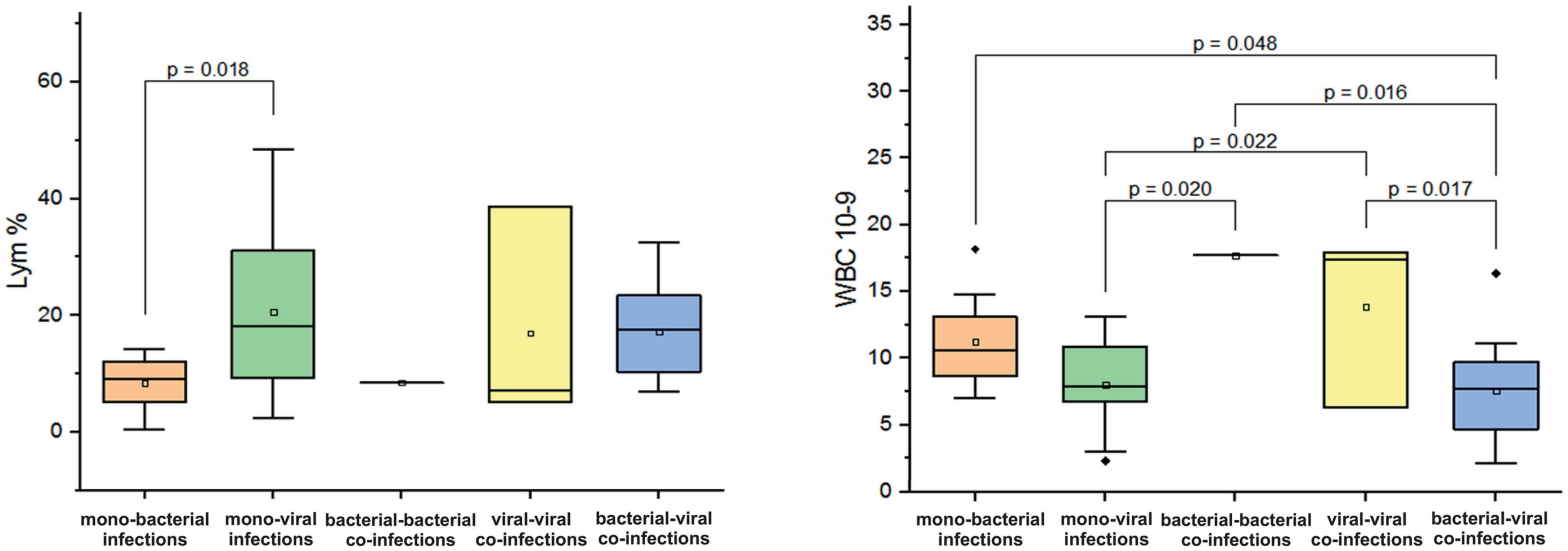
Comparison of the mean values of blood parameters: lymphocytes (Lym%) and leukocyte levels (WBC, 10^9/L) in children and adolescents aged 5–14 years with different categories of infections: mono-bacterial infections, mono-viral infections, bacterial-bacterial co-infections, viral-viral co-infections and bacterial-viral co-infections. Mean Lym% and WBC10^9/L values, median, and range 25–75% are shown (see Lym% and WBC10^9/L distribution). Values were calculated using the Mann–Whitney U test (▫ Mean; −Median Line; ┬Range within 1IQR; ♦ Outliers; ┐┌ Fisher LSD).

In children and adolescents aged 5–14 years, we observed an increase in leukocyte counts in cases of mono-bacterial infections (average WBC: 11.3 ± 3.6 × 10^9/L) and mixed viral-viral infections (average WBC 13.8 ± 6.5 × 10^9/L). In one case of confirmed bacterial-bacterial co-infection, the WBC count was = 17 10^9/L. In contrast, leukocyte counts for mono-viral and mixed bacterial-viral infections significantly remained within normal limits (normal WBC: 3.5–10.5 × 10^9/L, *p* < 0.05, Figure 6). Furthermore, we noted marked lymphocytopenia in cases of bacterial mono-infections (mean 8.4 ± 4.6%; *p* = 0.018, Figure 6) and bacterial-bacterial co-infections (mean 8.5%). In mono-viral, viral-viral, and bacterial-viral infections, Lym levels were either mild or remained within the normal range of 20–40% (mean values: 20.6 ± 13.5%; 16.9 ± 18.7%; 17.2 ± 8.4%). Furthermore, in all combinations of mono- and co-infections, we observed a three- to six-fold increase in CRP levels, exceeding the normal threshold of <5 mg/L.

#### 3.5.6 Age group 15–29 years

**Figure 7:**
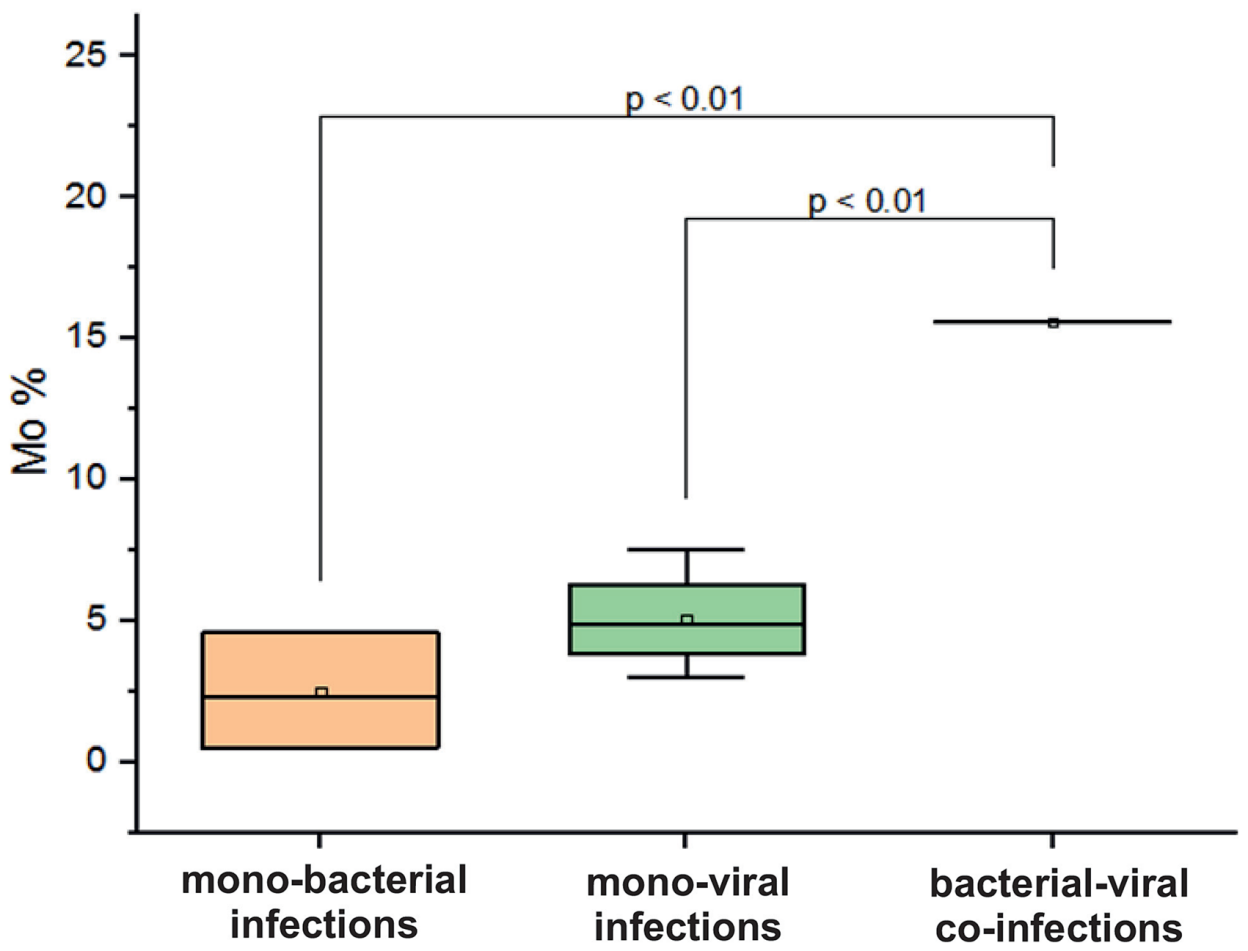
Comparison of mean values of blood parameter monocytes (Mo%) in adolescents and adults aged 15–29 years across different categories of infections: mono-bacterial infections, mono-viral infections, bacterial-bacterial co-infections, viral-viral co-infections, and bacterial-viral co-infections. Mean Mo% values, median, and range 25–75% are shown (see Mo% distribution). Values were calculated using the Mann–Whitney U test (▫ Mean; −Median Line; ┬Range within 1IQR; ♦ Outliers; ┐┌ Fisher LSD).

In adolescents and adults aged 15–29 years, a decrease in Mo was observed in cases of mono-bacterial infection, with a mean level of 2.4 ± 2% (normal range: 3.5–12.5%). In contrast, mixed bacterial-viral co-infection resulted in an increase in Mo by 15.6% (*p* = 0.01, Figure 7). In patients with confirmed viral mono-infections, a decrease in hemoglobin was observed, with a mean measured level of 108.3 ± 11.6 g/L (normal 120–180 g/L). In addition, a fivefold increase in measured mean CRP levels was documented in patients with monobacterial co-infections. However, in cases of monoviral and bacterial-viral mixed infections, CRP levels increased two to three times above the normal threshold of 5 mg/L.

#### 3.5.7 Age group 30–64 years

No significant differences were observed in various blood parameters in adult patients aged 30–64 years between groups of confirmed mono- and co-infections caused by respiratory pathogens. One patient in this age group had a mixed infection involving both bacterial and viral respiratory pathogens, which resulted in critical SpO2 levels of 89% and a significantly elevated CRP level of 171.9 mg/L. This patient also exhibited severe lymphocytopenia (Lym level: 7%) and monocytopenia (Mo level: 2%). In cases of mono-viral infections, a decrease in SpO2 was noted (mean: 91 ± 9.5%). The average CRP levels in these instances were recorded at 65.6 ± 132.8 mg/L, and monocytopenia showed a mean value of 2.3 ± 2.3%. For bacterial mono-infections, the only abnormal finding was in the CRP levels, which had a mean value CRP of 93 ± 99 mg/L.

#### 3.5.8 Age group <65 years

In a study involving patients aged <65 years, no significant differences were observed in blood parameter changes between those with mono-infections and those with co-infections. For monoviral infections and mixed infections (viral-viral and viral-bacterial), critical SpO2 and CRP levels were measured: mean SpO2 levels (87.4 ± 8.6%, 89.7 ± 8.6%, and 90 ± 5% for monoviral infections, viral-viral mixed infections, and viral-bacterial mixed infections, respectively); mean CRP levels (143.2 ± 103.4 mg/L, 150.8 ± 14.3 mg/L, and 206 ± 181.9 mg/L for monoviral infections, viral-viral mixed infections, and viral-bacterial mixed infections, respectively). In addition, leukocytosis and lymphocytopenia were observed, with average WBC counts of 12.2 ± 11.9 × 10^9/L and Lym percentages of 3.7 ± 2.6%. Reduced Mo levels were noted in patients with viral-viral mixed infections, with a mean Mo level of 1.9 ± 2.2%.

### 3.6 Clinical and laboratory characteristics of bacterial-viral co-infection and viral-viral co-infection

#### 3.6.1 In patients with confirmed mixed bacterial-viral infections, some blood parameter changes were observed

In children under 4 years of age, critical saturation levels were observed only in the 7–11-month age group, with a mean value of 89.6 ± 10.4%, while normal levels were measured in the other age groups (*p* < 0.05). In individuals over 65 years of age, saturation levels were lower (90 ± 5%) compared to those aged 2–4 years (*p* < 0.05). Notably, leukocytopenia was present in children aged 5–14 years with a mean leukocyte count of 7.5 ± 3.9 × 10^9/L, while leukocytosis was observed in infants aged 7–11 months and patients >65 years, with leukocyte counts of 12.6 ± 5.6 × 10^9/L and 13.58 ± 4.8 × 10^9/L, respectively (*p* < 0.05). Lymphocytopenia was observed in children and adolescents aged 5–14 years, with a higher incidence in young children aged 25–59 months compared to those <11 months (26.07 ± 20.16%, *p* < 0.05). Despite the pronounced lymphocytopenia in the age groups 7–11 months and 5–14 years, those over 30 years had relatively lower Lym levels than them (*p* < 0.05) (Table 7). In patients >64 years, the mean Mo levels measured in blood in % were lower compared to these changes in infants (*p* < 0.01). CRP levels were elevated in all groups, especially in older patients whose levels were five times above the normal range, or patients over 64 years of age had critically high CRP levels (206.6 ± 181.9 mg/L) compared with patients under 30 years of age (*p* < 0.05) (see Table 7).

**Table 8:** Distribution of the average measured values of blood parameters in patients with confirmed mixed viral-viral infections: saturation (SpO2%), white blood cells (WBC 10^9/L), lymphocytes (Lym%), monocytes (Mo%), hemoglobin g/L and C-reactive protein (CRP mg/L) in the following 8 age groups: 0-6 months, 7-11 months, 12-24 months, 25-59 months, 5-14 years, 15-29 years, 30-64 years, and under 64 years.

Age group	SpO2%, Mean	SpO2, SD	WBC 10^9/L, Mean	WBC 10^9/L, SD	Lym %, Mean	Lym %, SD	Mo %, Mean	Mo %, SD	Gran %, Mean	Gran %, SD	Hemoglobin g/L, Mean	Hemoglobin g/L, SD	CRP mg/L, Mean	CRP mg/L, SD
0–6 months	92.2	4.0	9.6	2.0	52.4	2.8	9.0	5.3	38.6	3.8	114.5	18.4	5.1	0.1
7–11 months	–	–	10.5	8.0	39.8	15.6	9.7	8.7	50.4	10.6	112.3	19.0	33.6	29.7
12–24 months	97.5	0.3	8.0	5.0	41.0	16.4	12.1	8.5	47.6	24.8	90.7	28.9	97.2	–
25–59 months	97.7	–	9.1	–	22.8	–	1.8	–	75.4	–	140.0	–	5.0	–
5–14 years	97.2	–	11.5	–	10.2	–	5.4	–	84.5	–	117.0	–	52.3	–
15–29 years	94.7	0.3	13.9	6.6	17.0	18.8	4.9	2.4	78.1	21.1	135.3	5.7	63.4	50.9
<64 years	89.7	8.6	14.0	11.4	3.8	2.6	1.9	2.2	94.4	0.4	133.0	5.7	150.8	14.3

#### 3.6.2 In patients with confirmed mixed viral-viral infections, some blood parameter changes were observed

In patients with confirmed viral-viral co-infection, lower saturation levels were observed only in those aged >64 years, with a mean value of 89.7 ± 8.6. For individuals in younger age groups, saturation levels were within normal limits. Elevated WBC counts were noted in patients aged >30 years with mean values of 11 ± 4 × 10^9/L and 13.9 ± 6.5 × 10^9/L, well above the normal range of 3.5–10.5 × 10^9/L. Normal Lym levels were recorded in all children aged <4 years. However, in two other age groups, 5–14 and 15–29 years, lymphocytopenia was observed with mean Lym levels of 10.2% and 16.9 ± 18.8%, respectively (compared with those aged <2 years, *p* < 0.05). Patients aged >65 years showed marked lymphocytopenia with a mean of 3.75 ± 2.6%, especially when compared with the mean levels in children aged <2 years (*p* < 0.05). Only patients aged >64 years and children aged 25–59 months showed decreased Mo levels with a mean of 1.8 ± 2.1%. The oldest patients also had higher Gran levels, with a mean of 94.4 ± 0.4%, significantly different from the mean Gran levels measured in children aged <2 years, *p* = 0.05. In these children and other age groups, gran levels remained normal (28–46% for 0–6 months and 30–80% for >6 months). In addition, lower mean hemoglobin levels were recorded in children aged 12–24 months compared with those aged 25–59 months, with a mean of 90.6 g/L versus 140 g/L (*p* = 0.044). Hemoglobin levels for the remaining age groups were within normal limits: 96–130 g/L for 0–6 months, 105–130 g/L for 7–11 months, and 120–180 g/L for adults. In infants aged 0–6 years, no CRP elevation was recorded, with levels remaining within the normal range of up to 10 mg/L. However, in children aged 12–24 months, a threefold increase above normal was observed, while children and adolescents aged 5–14 years showed a five-fold increase (mean CRP levels with 0–6 months 5 ± 0.1 mg/L vs. 33.6 ± 29.7 mg/L, *p* = 0.022; mean 52.3 mg/L, *p* = 0.031). Hospitalized patients >64 years with viral-viral mixed infections were characterized by critically elevated CRP levels, mean 150.8 ± 14.3 mg/L, compared with levels measured in children aged <15 years, *p* < 0.05 (Table 8).

## 4 Discussion

The COVID-19 pandemic has highlighted the need for a deeper understanding of the causes of progressive respiratory disease deterioration. Some studies have shown that co-infections involving both bacterial and viral agents may worsen COVID-19 severity (22). Before the pandemic, the risks of bacterial co-infections in patients with influenza were recognized but not systematically investigated (23). However, the effects of mixed viral infections and the role of bacterial-viral co-infections remain unclear. This study addresses this gap in the literature by identifying clinical-epidemiological features of respiratory infection caused by mono- and co-infections with various bacterial and viral pathogens. Using clinical and laboratory data, it also identified some clinicopathological features that may help distinguish bacterial from viral infections. A previous study conducted by our team focused specifically on patients who tested positive for SARS-CoV-2 and investigated the effects of bacterial-viral co-infections (7). This study extended this investigation to include cases of mono- and co-infections involving 14 respiratory viruses and 5 bacterial pathogens. A detection rate of 65.7% in our study was significantly higher than that in study conducted during the same period in China (34.7%) involving testing of 14 respiratory viruses (24). Two studies from 2013 and 2016 reported detection rates of at least one respiratory virus ranging from 30 to 45% using multiplex polymerase chain reaction testing of nasopharyngeal specimens (25, 26). In contrast, another study that examined 20 respiratory pathogens reported a rate of 65.9%, nearly identical to our findings (27).

In our study, we discovered that viral infections occurred more frequently than bacterial infections, which may be attributed to the higher proportion of children in our study, as RTIs are commonly caused by viral pathogens in children than in adults (28). Co-infections with respiratory pathogens were identified in 19.8–51.8% of positive respiratory samples across various studies, aligning with the findings of this study at 33% (29–31). The high detectability of SARS-CoV-2 can be attributed to an increase in its spread in Bulgaria during the spring–summer and autumn periods of this study. Our previous study reported the highest proportion of rhinoviruses (RV) among non-influenza respiratory viruses this finding also accounts for the significant number of identified mono- and co-infected patients with RV (32). In addition, studies have demonstrated that RV is the most frequently identified virus across nearly all age groups, particularly among young, infected individuals (31, 33).

We also observed a high percentage of mono- and co-infections with the bacterial pathogen *Streptococcus pneumoniae* (StPn), which is likely because it is normally present in the respiratory tract and is a primary LRTI causative agent (6, 34, 35). The elevated detection rate of these three respiratory pathogens further clarifies the high incidence of co-infections involving them. Moreover, evidence suggests that *S. pneumoniae* often contributes to mixed infections with SARS-CoV-2, consistent with our findings (36). Pneumococcal bacteremia is common in young children and can occur alongside illnesses such as meningitis, pneumonia, and septic arthritis, or be linked to localized infections such as acute otitis media. Approximately 3–5% of febrile children aged 3–36 months are at risk of asymptomatic bacteremia, with 85–95% of these cases being associated with *S. pneumoniae* before the introduction of the vaccine (37). In our study, we observed an increase in the incidence of *S. pneumoniae* infections among children aged 7 months to 5 years. The nasopharyngeal carriage of pneumococcal strains in healthy children is crucial in the horizontal transmission of these bacteria. This is particularly evident among children attending daycare centers (38). This study also concluded that mixed infections are more common in children aged <5 years, owing to the high prevalence of respiratory infections in this age group (39). Their less developed immune system makes them more susceptible to infections and allergic diseases than adults (40). Owing to their underdeveloped immune systems, particularly in newborns and infants up to 6 months old, children can carry the SARS-CoV-2 virus without displaying typical symptoms (41). This absence of symptoms may contribute to a higher occurrence of mixed infections, where SARS-CoV-2 is found alongside another viral or bacterial pathogen, as described in our study. In addition, a previous study of ours also reported a high rate of co-infections with SARS-CoV-2 among children aged 0–5 years (42). In other studies, AdVs have been identified as common co-pathogens, consistent with our findings (43, 44). AdV can infect various organ systems; however, most infections are asymptomatic (8). When combined with other bacterial and viral pathogens, they can lead to severe respiratory symptoms and result in hospitalization.

Bacterial and viral infection symptoms often overlap, making it difficult to distinguish between them (45). Sore throat is a common condition, usually caused by a viral or bacterial infection. However, our study found that people with sore throats were more likely to have a viral infection than a bacterial one (*p* < 0.05). Other studies also support the idea that viruses are a common cause of sore throats (46). However, *S. pyogenes* has been identified as the main causative agent. *S. pneumoniae* causes nonspecific gastrointestinal symptoms, such as nausea, vomiting, and diarrhea. We also found that diarrhea was more prevalent among patients infected with a bacterial pathogen compared with those with a viral infection (47). After analyzing the symptoms, we concluded that mixed infections involving three co-pathogens were more frequently detected after the tenth day following the onset of the first symptoms of respiratory infections. This finding indicates an increased likelihood of identifying mixed infections that include over two pathogens, particularly when a bacterial co-pathogen is involved. Secondary bacterial infections often occur 5–7 days, or even later, after the onset of symptoms from a primary viral infection (48, 49). The higher rates of detection of dual viral-viral infections in this group of patients support the evidence of dual mixed infections by the 10th day of illness. The mechanisms behind co-infection are complex; research suggests that viral changes in the respiratory tract may increase susceptibility to bacterial infections (48). Secondary bacterial infections may be facilitated by the cytopathic effects of the virus and the immune impairment resulting from inflammatory cytokine overproduction (3). Immune response changes can reduce the ability of the host to clear bacteria or increase inflammation, worsening the infection (50). Studies suggest that influenza can predispose individuals to bacterial pneumonia, with the timeframe for bacterial infections ranging from 7 to 21 days, although shorter periods have been observed during pandemics (51).

In this study, we identified SARS-CoV-2 as a significant pathogen associated with severe complications that can lead to hospitalization and, in some cases, death. In patients aged >65 years with pre-existing health conditions, COVID-19 frequently results in fatal outcomes (52). The presence of mixed infections may further exacerbate their condition (53, 54). In a previous study, as well as in this one, we reported a case of a patient infected with both SARS-CoV-2 and AdV (40). In addition, other studies have shown that patients with COVID-19 who with secondary bacterial infections have an increased risk of mortality (55, 56). This finding highlights the importance of annual revaccination to reduce the prevalence and risk of complications associated with respiratory viral infections and emphasizes the need for vaccination against some clinically significant bacterial pathogens that cause respiratory infections. In Bulgaria, the *Streptococcus pneumoniae* vaccine is mandatory and is administered in three booster doses after the 6th week of life. However, we observe a high percentage of children aged <5 years who are infected with *Streptococcus pneumoniae*. Current vaccines cover only a small part of the >100 different pneumococci serotypes (57). Currently, a 10-valent vaccine is used in Bulgaria, while a 25-valent vaccine is available on the market, which covers most of the common capsular serotypes of *Streptococcus pneumoniae*. In addition, serotype 3 is a significant cause of severe respiratory infections (Calvo-Silveria S). In our study, this serotype, along with the recently emerged 11A/D, demonstrated serious clinical severity, indicating a need to switch to the approved 20 or 23-valent vaccine, which includes both serotypes. The case fatality rate increases with age: 4% in children aged <15 years, 6% in those aged 15–44 years, 11% in individuals aged 45–64 years, and 21% in people aged >65 years (58). This finding is consistent with deaths associated with *Streptococcus pneumoniae* serotype 11A/D infection in patients aged >65 years, highlighting the need for a re-vaccination as these patients are at greater risk of complications and fatal outcomes. This risk is particularly pronounced in cases where a secondary bacterial infection follows a previous viral infection (59).

Bacterial and viral infections exhibit different clinical manifestations in different age groups. In infants and children <5 years of age, viral infections tend to result in more severe illness than in adults (60, 61). In contrast, SARS-CoV-2 shows a different pattern; children tend to experience milder symptoms than adults (62, 63). Newborns receive immunity from their mothers, which helps ensure a milder course of illness than other age groups (64). This observation is consistent with the mild changes in blood parameters observed in confirmed bacterial and viral mono- and co-infected infants. In children >7 months of age, lower mean SpO2 levels have been observed in cases of bacterial-viral infections, along with a threefold increase in CRP, indicating a more severe infection. Studies have shown that when CRP levels rise above 50 mg/L, there is a 90% probability that the infection is bacterial (65). Therefore, these findings suggest that in cases of bacterial-viral coinfections, in this study, the mean CRP levels found were more consistent with viral infection than with bacterial infection. Some mixed viral infections can lead to significant elevations in CRP levels, especially in cases of superinfection (66). This increase is particularly notable in cases involving confirmed mixed infection with SARS-CoV-2 (42, 67). Furthermore, the most significant increases in mean CRP levels have been observed in patients aged >65 years, and in bacterial-viral infections, CRP levels can exceed 200 mg/L. Elevated CRP levels, combined with lymphocytopenia and decreased oxygen saturation, commonly seen in co-infected patients, are more likely to indicate severe COVID-19 (68), making the presence of bacterial or viral co-infection a particular risk factor in patients aged >65 years.

In our study of children under 14 years of age, the saturation levels in cases of viral-viral mixed infections remained within normal limits, whereas those in bacterial-viral mixed infections were critically low. These findings suggest that co-infection with a bacterial pathogen is an aggravating factor and that these copathogens may negatively affect clinical and pathological outcomes. This study emphasizes the need to pay special attention to the need to exclude viral infection in the presence of bacteria, especially when bacteria are considered normal findings in the nasopharynx. Evaluating the risks of viral-bacterial co-infections in hospitalized patients with influenza or other respiratory viruses can assist clinicians in managing the morbidity and mortality associated with bacterial infections. This assessment may promote the broader adoption of multiplex PCR diagnostics, which can simultaneously detect a wide range of viral and bacterial pathogens, particularly in patients facing severe clinical outcomes. Negative results from traditional methods used to diagnose bacterial pathogens should not rule out the possibility of a secondary or co-infection with a bacterial pathogen in patients experiencing respiratory symptoms. Rapid and precise identification of the cause of the respiratory infection would enhance clinical and pathological assessments, ultimately contributing to more favorable clinical outcomes. Although this study reached important conclusions, we must acknowledge its limitations. The study duration was insufficient, preventing the inclusion of a larger proportion of patients with a positive influenza test. Moreover, we examined only five respiratory bacterial pathogens, which do not fully represent the potential for co-infections. In addition, it should be noted that additional clinical materials, such as sputum were not used for the study, and combining nasopharyngeal and sputum samples would have provided more complete results in terms of the presence of additional bacterial pathogens. Future studies should address these limitations by expanding the study to include a wider variety of bacterial pathogens and monitoring their antibiotic resistance. This approach will improve understanding of the role of viral-viral and bacterial-viral mixed infections in the progressive deterioration of respiratory disease.

In conclusion, this study identified SARS-CoV-2, RV, AdV, and *S. pneumoniae* as key respiratory pathogens responsible for a significant number of mono- and co-infections. Notably, cases of mixed infections involving SARS-CoV-2 were associated with symptom worsening, particularly in patients aged >65 years. Therefore, expanding the diagnostic approach to include a broader spectrum of bacterial and viral pathogens will facilitate early and targeted treatment. Further, this study highlights the need for preventive measures, including vaccination, and strongly recommends the expansion of available vaccine types. In addition, older adults should be re-vaccinated with a vaccine that encompasses a broader range of *S. pneumoniae* serotypes.

## Data Availability

The original contributions presented in the study are included in the article/Supplementary material, further inquiries can be directed to the corresponding author.

## Footnotes

^1^ https://www.graphpad.com/quickcalcs/contingency1
