# Supplementary material for: Respiratory infections in the post-COVID-19 era: impact, prevalence, and clinical characteristics of bacterial and viral co-infections
Source: Front Med (Lausanne). 2025 Oct 23;12:1597782. doi: 10.3389/fmed.2025.1597782 (PMC12589918; doi:10.3389/fmed.2025.1597782)
Supplement: Supplementary file 1 [file Table_1.docx]

|  | **Norm of blood parameters according to the age of the patients** | | |
| --- | --- | --- | --- |
| **Indicator** | **0–6 months** | **7–11 months** | **> 12 months and adults** |
| **Blood oxygen saturation (SpO2, %)** | 93-100 | 93-100 | 95-100 |
| **Lymphocytes (Lym, %)** | 37-73 | 20-40 | 20-40 |
| **Monocytes (Mo,%)** | 1-6 | 3.5-12.2 | 3.5-12.2 |
| **Granulocytes (Gr,%)** | 28–46 | 50-80 | 50-80 |
| **Hemoglobin (Hem, g/L)** | 96–130 | 105-130 | 120-180 |
| **Leukocytes (WBC, ×10⁹/L)** | 6-17 | 6-17 | 3.5-10.5 |
| **C-reactive protein (CRP, mg/L)** | 0-10 | 0-5 | 0-5 |

Supplementary table 1: Norm of blood parameters: blood oxygen saturation (SpO2, %) lymphocytes (Lym %), monocytes (Mo %), granulocytes (Gr %), hemoglobin (Hem g/L), leukocytes (WBC 10⁹/L), C-reactive protein (CRP mg/L) according to the age group of the patients.
