# Supplementary material for: Respiratory infections in the post-COVID-19 era: impact, prevalence, and clinical characteristics of bacterial and viral co-infections
Source: Front Med (Lausanne). 2025 Oct 23;12:1597782. doi: 10.3389/fmed.2025.1597782 (PMC12589918; doi:10.3389/fmed.2025.1597782)
Supplement: Supplementary file 2 [file Table_2.docx]

| **Pathogen** | **Gen** |  | **Primer nucleotide sequence** | nM |
| --- | --- | --- | --- | --- |
| S. pneumoniae | *lytA* | F | ACGCAATCTAGCAGATGAAGCA | **250** |
|  |  | R | TCGTGCGTTTTAATTCCAGCT | **250** |
|  |  | P | HEX–TGCCGAAAACGCTTGATACAGGGAG | **100** |
| Streptococcus  pyogenes | *spy* | F | GCACTCGCTACTATTTCTTACCTCAA | **300** |
|  |  | R | GTCACAATGTCTTGGAAACCAGTAAT | **300** |
|  |  | P | HEX-CCGCAACTCATCAAGGATTTCTGTTACCA | **100** |
| Chlamydophila  pneumoniae | *ompA* | F | GGGCTATAAAGGCGTTGCTTT | **500** |
|  |  | R | AGACTTTGTTCCAGTAGCTGTTGCT | **500** |
|  |  | P | Texas Red-CCTTGCCAACAGACGCTGGCG | **200** |
| H. influenzae | *bexA* | F | TGCGGTAGTGTTAGAAAATGGTATTATG | **600** |
|  |  | R | GGACAAACATCACAAGCGGTTA | **600** |
|  |  | P | FAM-ACA AAG CGT ATC AA“T”ACTACAACGAGACGC AAAAA3 | **100** |
| Mycoplasma pneumoniae | *ATPase* | F | AAGAAGCTTATGGTACAGGTTGGTTAA | **300** |
|  |  | R | TGGAGGTTGGTAGCTAAGTAAGCA | **900** |
|  |  | P | FAM-TGACTGGAAGGA “T“GTTAAGCAGGACAACAA ATTT | **150** |

Supplementary table 2: Nucleotide sequences of the primers and probes used for detection of: S. pneumoniae, S. pyogenes, Ch. pneumoniae, H. influenzae and M. pneumoniae.
